# Comparison of bronchoscopic and non-bronchoscopic techniques for diagnosis of ventilator associated pneumonia

**DOI:** 10.4103/0972-5229.78218

**Published:** 2011

**Authors:** G. C. Khilnani, T. K. Luqman Arafath, Vijay Hadda, Arti Kapil, Seema Sood, S. K. Sharma

**Affiliations:** **From:** Department of Medicine, All India Institute of Medical Sciences, New Delhi, India; 1 Department of Microbiology, All India Institute of Medical Sciences, New Delhi, India

**Keywords:** Bronchoscopic brush, bronchoalveolar lavage, endotracheal aspirate, non-bronchoscopic bronchoalveolar lavage, ventilator associated pneumonia

## Abstract

**Background:**

The diagnosis of ventilator associated pneumonia (VAP) remains a challenge because the clinical signs and symptoms lack both sensitivity and specificity and the selection of microbiologic diagnostic procedure is still a matter of debate.

**Aims and Objective:**

To study the role of various bronchoscopic and non-bronchoscopic diagnostic techniques for diagnosis of VAP.

**Settings and Design:**

This prospective comparative study was conducted in a medical ICU of a tertiary care center.

**Materials and Methods:**

Twenty-five patients, clinically diagnosed with VAP, were evaluated by bronchoscopic and non-bronchoscopic procedures for diagnosis. The sensitivity, specificity, positive predictive value (PPV) and negative predictive value (NPV) of various bronchoscopic and non-bronchoscopic techniques were calculated, taking clinical pulmonary infection score (CPIS) of ≥6 as reference standard.

**Results:**

Our study has shown that for the diagnosis of VAP, bronchoscopic brush had a sensitivity, specificity, PPV and NPV of 94.9% [confidence interval (CI): 70.6–99.7], 57.1% (CI: 13.4–86.1), 85% (CI: 61.1–96) and 80% (CI: 21.9–98.7), respectively. Bronchoscopic bronchoalveolar lavage (BAL) had a sensitivity, specificity, PPV and NPV of 77.8% (CI: 51.9–92.6), 71.8% (CI: 24.1–94), 87.3% (CI: 60.4–97.8) and 55.5% (CI: 17.4–82.6), respectively. Sensitivity, specificity, PPV and NPV for non–bronchoscopic BAL (NBAL) were 83.3% (CI: 57.7–95.6), 71.43% (CI: 24.1–94), 88.2% (CI: 62.3–97.4) and 62.5% (CI: 20.2–88.2), respectively. Endotracheal aspirate (ETA) yield was only 52% and showed poor concordance with BAL (κ-0.351; P-0.064) and NBAL (k-0.272; *P*-0.161). There was a good microbiologic concordance among different bronchoscopic and non-bronchoscopic distal airway sampling techniques.

**Conclusion:**

NBAL is an inexpensive, easy, and useful technique for microbiologic diagnosis of VAP. Our findings, if verified, might simplify the approach for the diagnosis of VAP.

## Introduction

The diagnosis of ventilator associated pneumonia (VAP)remains a challenge because the clinical signs andsymptoms lack both sensitivity and specificity and the selectionof microbiologic diagnostic procedure is still a matter of debate.[1] Clinically, VAP is defined byfour criteria: (1) the radiographic appearance of a new or progressivepulmonary infiltrates, (2) fever, (3) leukocytosis, and (4) purulenttracheobronchial secretions.[2] However, each of these signs or symptoms taken separately has limited diagnostic value and may also be seen in a noninfectious process.[3,4] Pugin and colleagues combinedbody temperature, white blood cells count, volume and appearanceof tracheal secretions, oxygenation (PaO _2_ /FiO _2_), chest X-ray, and tracheal aspirate cultures into a clinical pulmonary infectionscore (CPIS) as a diagnostic tool for VAP and foundthat a CPIS of >6 was associated with a high likelihoodof pneumonia with a sensitivity and a specificity of93 and 100%, respectively.[5] Subsequently, Singh and colleagues validated this finding by using CPIS score successfully in reducing unnecessary antibiotic use in patients in whom VAP wassuspected.[6]

Accurate clinical and microbiologic diagnosis of VAP is essential not only for selection of appropriate antimicrobials but also to prevent their misuse. Inappropriate use of antimicrobials in these patients leads to increased mortality and emergence of multidrug resistant pathogens in the ICU.[7] It has been postulated by numerous investigatorsthat “invasive” diagnostic methods, including quantitativecultures of distal airway specimens obtained by using bronchoscopic bronchoalveolarlavage (BAL), bronchoscopic brush, protected BAL, or protected specimen brush (PSB), could improve identificationof patients with true VAP and selectionof appropriate antibiotics.[8–10] However, bronchoscopy requires technical expertise and adds to the cost of care. The results of the studies using bronchoscopic techniques are inconsistent, showing both false-positive and false-negative results, which further questions their exact role in the diagnosis of VAP.[11,12] In an attempt to overcome these limitations, non-bronchoscopic distal airway sampling methods have emerged, like non-bronchoscopic BAL (NBAL) and non-bronchoscopic PSB. Though simple and inexpensive, diagnostic accuracy of these blind sampling methods has not been studied in the Indian setting.

This study was designed to compare the diagnostic value of various methods of collecting respiratory samples which included bronchoscopic BAL and brushings, non-bronchoscopic protected BAL, and endotracheal aspirate (ETA) in patients with VAP.

## Materials and Methods

This prospective comparative study was conducted in the medical intensive care unit (ICU) of a tertiary care center situated in northern India. The study was approved by the institutional review board. After obtaining written informed consent from either the patient or the first-degree relative, we enrolled 25 patients of age 18 years or more who required ventilatory support for the preceding 48 hours or more with clinical and radiological diagnosis of VAP.[13] Patients with diagnosis of community acquired pneumonia, immunocompromised state and bleeding diathesis were excluded. Similarly, patients who required mechanical ventilator in another hospital for 48 hours or more before admission to our institute and those who were extubated/weaned or died within 3 days of intubation were also excluded from the study. For each patient studied, these parameters wererecorded: age, gender, primary diagnosis on admission, indication of mechanical ventilation, comorbid conditions (chronic obstructive airway disease, alcoholism, cardiac or neurological disease), Glasgow coma score, organ failure and severity scores assessedby APACHE II, duration of mechanical ventilation, and antibioticsreceived before samplings.

In each patient, four respiratory samples were collected which included ETA, bronchoscopic brush and BAL, and NBAL. To avoid contamination of the lower airways, the non-bronchoscopic sampling was performed first. The samples were obtained in sequential order of ETA followed by NBAL, then bronchoscopic brush and BAL. All the bronchscopic samplings were done by the principal investigator (GCK) and non-bronchoscopic procedures by a junior resident doctor.

### 

#### Endotracheal aspirate and non-bronchoscopic BAL

**Figure 1:**
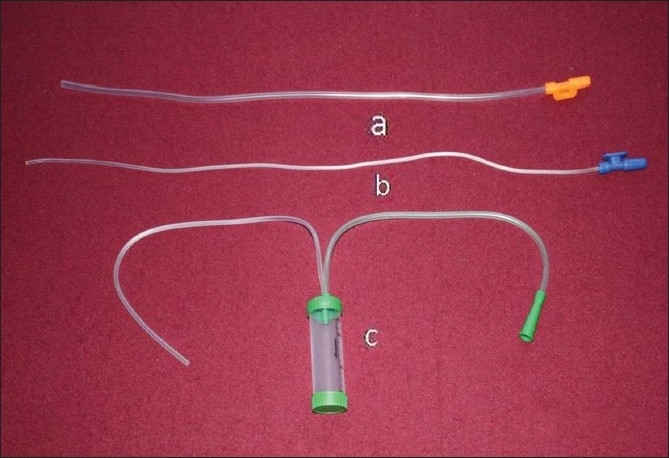
The apparatus used for non-bronchoscopic lavage. It comprises two suction catheters (a, b) and one mucus trap (c). The suction catheters have different lengths and luminal diameters. The upper suction catheter (a) is shorter in length (about 47–48 cm) and with wider lumen (16 Fr). The second catheter (b) is longer (50 cm) and with narrower lumen (8 Fr)

The ETA specimens werecollected via a sputum suction trap. Non-bronchoscopic protected BAL was performed by double catheter technique. A sterile suction catheter of size 16 Fr was cut 2–3 cm from the distal end to give a final length of about 47–48 cm and inserted through the endotracheal tube and blindly advanced into the distal airways till resistance is felt. The catheter was wedged in that position, and a second, 50-cm long, sterile suction catheter of size 8 Fr was passed through the first catheter and advanced as far as possible. Twenty milliliters of normal saline was instilled into the distal airways through the inner tube and aspirate was collected in a sterile container [Figure 1]. Quantity of the aspirate was recorded. Procedure was repeated if the aspirated fluid was less than 5 ml. The samples were immediately transported for bacteriologic examination and quantitative cultures.

#### Bronchoscopic BAL and brush

Patients were sedated with 5 mg of intravenous midazolam. The ventilatory settings were adjusted by increasing tidal volume by 100 ml and FiO _2_ to 1.0. All the vitals including heart rate, blood pressure and oxygen saturation were monitored using continuous pulse oxymetry during the entire procedure.

The bronchoscope was introduced through the T-piece and the tip was positioned close to the orifice of the bronchus, draining the bronchopulmonary segment of interest as determined by chest radiograph. In patients with diffuse/bilateral lung infiltrates, bronchoscope was advanced into a bronchopulmonary segment of the right lower lobe for sampling. On reaching the area to be sampled, the brush was protruded, plunged a number of times into the suspected bronchus, and withdrawn. The sample was then transferred by stirring the brush into a sterile vial containing 1 ml of normal saline which was used for microbiologic analysis.

Bronchoscopic BAL was performed in the same lobe and segment/sub-segment from which bronchoscopic brushings were obtained. After introducing the bronchoscope and wedging the tip in the selected segmental or sub-segmental bronchus, at least three aliquots of 25 ml buffered normal saline were instilled and gently aspirated by suction.

All the samples were transported to our microbiology laboratory within 1 hour of collection and further processing was done there as described below.

BAL fluid – Samples were divided into two, the first half was centrifuged (1500 rpm/minute for 10 minutes) and used for gram stain. The second half of the BAL sample was serially diluted with normal saline (1:10–1:10^6^) and used for quantification of bacterial load.

Bronchial brushing specimens – The samples were vortexed vigorously for 60 seconds to thoroughly suspend all the materials from the brush into the saline solution. This was examined microscopically following gram stain. Rest were serially diluted similar to BAL and used for culture.

ETA – ETA samples were used directly for staining and microbiologic culture by semi-quantitative method.

Ten μl of each dilution was transferred on to dry blood agar and Mc Conckey agar plate and spread properly. The plates were incubated at 35–37°C for 24 hours. Bacterial identification was done using standard microbiologic techniques and antibiotic sensitivity was estimated as per National Committee for Clinical Laboratory Standard (NCCLS). The growths were expressed as number of colony forming units (CFU)/ml. Thethresholds applied to quantitative cultures for thediagnosis of VAP were 10^3^ CFU/ml for bronchoscopic brush sample and 10^4^ CFU/ml for non-bronchoscopic protected BAL and bronchoscopic BAL. ETA samples were cultured semi-quantitatively and were considered positive only when the same organism was isolated from other distal sampling techniques with their respective cut-off values.

The sensitivity, specificity, positive predictive value (PPV) and negative predictive value (NPV) of non-bronchoscopic protected BAL, bronchoscopic BAL and bronchoscopic brushings, for the diagnosis of VAP, were calculated taking CPIS score of 6 as reference standard. A kappa (κ) coefficient of agreement among these three diagnostic modalities was computed. Results were presented as mean ± SD and a *P* value of <0.05 was considered significant.

## Results

We prospectively evaluated 25 patients with high clinical suspicion of VAP, between August 2002 to March 2004. General characteristics of the patients are shown in Table 1. There was a male preponderance in the study group with a male:female ratio of 17:8. Chronic obstructive pulmonary disease with type-II respiratory failure (14) was the commonest indication of mechanical ventilation. Other diseases include metabolic encephalopathy (2), cerebral vascular disease (2), acute inflammatory demyelinating polyneuropathy (1), acute disseminated encephalomyelitis (1), chronic liver disease (1), myotonic muscular dystrophy (1), organophosphorus poisoning (1), chronic renal failure with anemia (1), and sub-dural hematoma due to road traffic accident (1). Majority of patients, except two cases, were diagnosed to have late onset VAP. All patients in the study were on presumptive antibiotic treatment.

**Table 1:** Baseline characteristics of patients

Variables	Results [Mean ± SD (range)]
Age (years)	55.60 ± 16.17 (24–80)
Duration of ICU stay (days)	29.52 ± 24 (9–106)
Duration of mechanical ventilation (days)	34.88 ± 32 (7–98)
Duration of development of VAP (days)	18.44 ± 12.18 (4–57)
Duration of fever (days)	4.56 ± 3.14
Duration of increased tracheal secretion (days)	5.36 ± 2.18
Leukocyte counts of the cohort (per mm^3^)	12,447 ± 3592
pO_2_ of cohort (mm of Hg)	90 ± 19.18
pCO_2_ of cohort (mm of Hg)	47.23 ± 25.12
CPIS of cohort	6.76 ± 1.67
APACHE II	23 ± 7.12

CPIS – clinical pulmonary infection score

There was no significant complication observed during or immediately after the sampling procedures. Overall mortality was 60%. No patient deaths were attributed to sampling techniques, either bronchoscopic or non-bronchoscopic.

Four samples (non-bronchoscopic protected BAL, bronchoscopic BAL, bronchoscopic brush and ETA) from each patient with high clinical suspicion of VAP were analyzed. The diagnostic utility of various sampling techniques is shown in Table 2. The yields of different sampling techniques were 80% for bronchoscopic brush, and 64 and 68% for bronchoscopic BAL and non-bronchoscopic protected BAL, respectively. For ETA, the samples’ yield was only 52%.

**Table 2:** Diagnostic value of various sampling techniques

Sampling techniques	Yield	Sensitivity (95% CI)	Specificity (95% CI)	PPV (95% CI)	NPV (95% CI)
ETA	52	55.6 (31.3–77.6)	71.4 (30.3–94.9)	83.3 (50.9–97.1)	38.5 (15.1–67.7)
NPBAL	68	83.3 (57.7–95.6)	71.4 (24.1–94.0)	88.2 (62.3–97.4)	62.5 (21.5–88.2)
BAL	64	77.8 (51.9–92.6)	71.8 (24.1–94.0)	87.3 (60.4–97.8)	55.5 (17.4–82.6)
B Brush	80	94.9 (70.6–99.7)	57.1 (13.4–86.1)	85 (61.1–96.0)	80 (21.9–98.7)

NPBAL – Non-bronchoscopic protected bronchoalveolar lavage; B brush – Bronchoscopic brush; ETA – Endotracheal aspirate; PPV – Positive predictive value; NPV – Negative predictive value

A definite clinical diagnosis of VAP, i.e., a CPIS score of ≥6, was present in 18 patients. Non-bronchoscopic protected BAL had a kappa measure of agreement of 0.525 (*P*-0.017) with the clinical diagnosis of VAP. Sensitivity, specificity, PPV and NPV were 83.3% (CI: 577–7.6), 71.43% (CI: 241–1), 88.2% (CI: 623–3.4) and 62.5% (CI: 202–2.2), respectively. Percentage of concordance between non-bronchoscopic protected BAL and CPIS was 80%.

In our study, bronchoscopic brush samples showed the highest agreement with CPIS, with a kappa value of 0.565 (P-0.012). It has shown a sensitivity of 94.9% (CI: 706–6.7) with specificity of 57.1% (CI: 134–4.1). PPV and NPV were 85% (CI: 611–1) and 80% (CI: 219–9.7), respectively. Bronchoscopic BAL had a sensitivity of 77.8% (CI: 519–9.6) and specificity of 71.8% (CI: 241–1). PPV and NPV were 87.3% (CI: 604–4.8) and 55.5% (CI: 174–4.6), respectively. The kappa measure of agreement with CPIS was 0.453(*P*-0.021) and percentage of concordance was 76%.

Among the different sampling techniques, we observed highest concordance for the type of microorganisms between non-bronchoscopic protected BAL and bronchoscopic brush. This was followed by non-bronchoscopic protected BAL and bronchoscopic BAL and two bronchoscopic techniques, BAL and brush [Table 3]. Semi-quantitative ETA culture showed low concordance rate with bronchoscopic (BAL and brush) as well as non-bronchoscopic techniques (BAL). However, it showed highest and statistically significant microbiologic concordance with bronchoscopic brush.

**Table 3:** Concordance among various microbiologic sampling techniques

Sampling techniques	Kappa coefficient	% Concordance	*P* value
NPBAL vs. B brush	0.694	88	0.001
BAL vs.B brush	0.423	76	0.04
NPBAL vs. BAL	0.56	80	0.01
ETA vs.NPBAL	0.351	68	0.064
ETA vs.BAL	0.272	64	0.161
ETA vs. B brush	0.426	72	0.009

NPBAL – Non-bronchoscopic protected bronchoalveolar lavage; B brush – Bronchoscopic brush; ETA – Endotracheal aspirate

Microbial cultures were positivein 21 of 25 (84.0%) samples each of bronchoscopic brush and BAL. Non-bronchoscopic protected BAL and ETA samples grew pathologic microorganisms on 22 (88%) and 17 (68%) samples cultured, respectively. *Pseudomonas aeruginosa* was the most common organism isolated (44 of 100 samples), followed by Acinetobacter (37%), *Escherichia coli* and *Klebseilla* [Table 4]. Among these pathogens, 90% were extended spectrum beta-lactamase (ESBL) positive. Average number of organisms present per sample in ETA and non-bronchoscopic protected BAL were 0.88 and 1.28, respectively. For BAL and bronchoscopic brush, the numbers of organism in each sample were similar, i.e., 1.33 each. Perfect qualitative concordance (organism and antibiotic sensitivity) among all four techniques was seen in 9 out of 25 cases. When distal airway sampling techniques (non-bronchoscopic protected BAL, BAL and bronchoscopic brush) alone were considered, this increased to 21 out of 25.

**Table 4:** Organisms isolated in microbiologic cultures

Organisms	Various respiratory samples [n (%)]			
	B. Brush	BAL	NPBAL	ETA
Sterile	4(16)	4(16)	3(12)	8 (32)
Pseudomonas aerugenosa (ESBL+)	6(24)	6(24)	6(24)	7 (28)
Pseudomonas aerugenosa (ESBL–)	1(4)	1(4)	1(4)	1 (4)
Pseudomonas aerugenosa (ESBL–)and Acinetobacter spp.(ESBL+)	1(4)	1(4)	1(4)	Nil
Enterobacter spp.(ESBL+) and Pseudomonasaerugenosa (ESBL+)	1(4)	1(4)	1(4)	Nil
Klebsiellaspp. (ESBL–)	1(4)	1(4)	1(4)	Nil
Acinetobacter spp.(ESBL+) and Escherichia coli (ESBL+)	1(4)	1(4)	2(8)	Nil
Escherichia coli (ESBL+)	2(8)	2(8)	2(8)	3 (12)
Acinetobacter spp.(ESBL+)	5(20)	5(20)	5(20)	4 (16)
Pseudomonas aerugenosa (ESBL+)and Acinetobacter spp.(ESBL+)	3(12)	3(12)	3(12)	Nil
Acinetobacter spp.(ESBL–)	Nil	Nil	Nil	1 (4)
Acinetobacterspp. (ESBL+) and Citrobacter (ESBL+)	Nil	Nil	Nil	1 (4)

B. brush – Bronchoscopic brush; BAL – Bronchoalveolar lavage; NPBAL – Non-bronchoscopic protected bronchoalveolar lavage; ETA – Endotracheal aspirate; ELBS – Extended spectrum beta-lactamase

## Discussion

VAP is a common complication associated with invasive ventilator support and contributes to a significant mortality and morbidity in these patients.[14,15] Because of poor specificity of the clinical diagnosis of VAP, reliance is often placed on radiologic and microbiologic diagnosis. Microbiologic diagnosis comprises culture of blood, pleural fluid and respiratory secretions including proximal (tracheal aspirate) and distal airways (BAL and brush). It is important to keep in mind that the sensitivity of blood culture for diagnosis of VAP is less than 25%, and even when positive, the organism may originate from an extrapulmonary site of infection in as many as 64% of cases, even when VAP is present.[16]

ETA is the most commonly used method of airway sampling in ICUs all over the world. Gram stain, non-quantitative and semi-quantitative culture of tracheal secretions has the advantage of reproducibility and of requiring little technical expertise and no specialized equipment or technique. However, these studies add little to the sensitivity and specificity of the clinical diagnosis of VAP, as the upper respiratory tract is frequently colonized with potential pathogens, even in the absence of pneumonia.[12,17] Thus, if an organism is cultured or noted on gram stain, one does not know if it is the cause of pneumonia or simply colonization. Our study too questioned the value of microbiologic diagnosis of VAP based on ETA culture results by demonstrating its poor microbiologic agreement with distal airway sampling techniques. It highlights that ETA does not represent a true distal airway sample. Therefore, treating VAP based on ETA microbiology may not be the optimum management of these patients.

Studies from various countries have shown that bronchoscopic procedures are important part of evaluation of patients with VAP. However, these are associated with false-positive and false-negative results.[18–20] Canadian Critical Care Trial group, in a large multicentric study, demonstrated that there was no difference in clinical outcome among patients treated for VAP based on bronchoscopic or non-bronchoscopic procedures.[21] Further, in developing countries like India, this facility is not routinely available for patients admitted to ICU. Therefore, it is important to evaluate the role of non-bronchoscopic techniques in our setting. Our study has shown that NBAL has good sensitivity, specificity, PPV and NPV. Inherent advantages of non-bronchoscopic techniques include less invasiveness, lesser compromise of oxygenation, ventilation and respiratory mechanics during the procedure, less likelihood of increasing intracranial pressure and arrhythmia, lack of contamination through the bronchoscopic channel, and lower cost.

Although NBAL is a blind procedure, its concordance with bronchoscopic brush proves the fact that protected sample adequately represents the lower airway secretions (either side) and efficiently diagnoses VAP. The utility of NBAL for diagnosis of VAP has been demonstrated by other researchers also, both in clinical as well as autopsy studies.[5,22] Rouby *et al*. showed that the sensitivity and specificity of NBAL were 70 and 69%, respectively, using post-mortem histologic and bacteriologic analysis of lung as the gold standard for the diagnosis of VAP.[22] Pugin *et al*. used CPIS as the diagnostic criteria for VAP and found that sensitivity, specificity, and PPV of non-bronchoscopic BAL were 73, 96, and 92%, respectively.[5] Many other researchers also have shown that NBAL has high sensitivity (70–70%) and specificity (69–69%) depending on the criteria used to diagnose VAP.[5,23–25] Our study results are comparable to these and we hope that this technique would find utility in clinical practice.

We observed a good agreement for the type of microorganisms among bronchoscopic BAL, brush and non-bronchoscopic BAL. The predominant pathogens cultured were identical in 84% of samples. Kollef *et al*. in their study showed that NBAL done by a respiratory physiotherapist has shown good microbiologic agreement (83.3%) with bronchoscopic protected brush.[24] These results signify that blind sampling techniques like NBAL are good modalities for microbiologic diagnosis of VAP. As shown in another recent trial,[26] our study also highlights that ETA does not represent a true distal airway sample. The concordance of NBAL with bronchoscopic brush proves the fact that protected sample adequately represents lower airway secretions (either side) and accurately diagnoses VAP.[5,13,22,24] Therefore, ETA should be replaced by NBAL for microbiologic diagnosis of VAP.

Theoretically, there may be concern of diluting the alveolar fluid for the bronchoscopic samples when invasive samples (BAL and brush) are obtained after the non-bronchoscopic samples (NBAL). However, most of the studies have followed this protocol and did not find significant effect of this dilution on microbiology of the sample.[13,23,24,26,27] This effect will be further nullified by setting different thresholds for CFU/ml for each microbiologic sample, such as 10^3^ for brush and 10^4^ for BAL.

An important consideration in our study is the financialimplications associated with providing these diagnostic procedures. Bronchoscopic procedures are expensive, require expertise and are not freely available, whereas NBAL is a simple procedure which can be performed by resident doctors and paramedics (nurses) posted at the ICU after a small demonstration. This will result in significant reduction in the cost of management of VAP as shown in a report by Kollef *et al*., where about $1000 was saved per substitution of bronchoscopic brush with NBAL.[24] Similar benefits should be expected in our setting as the catheters and mucus extractor used in our study cost only 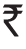
50 per patient. However, total savings depend both on these direct savings andon the balance of false-positive and false-negative resultsproduced by these tests with their ensuing costs and patientcharges.

An important limitation of our study is the validity of the exact operating characteristics (sensitivity, specificity, PPV, and NPV) for various sampling techniques, which may be questioned in the absence of the gold standard for the diagnosis of VAP. Autopsy examination of lung tissue (bacteriologic and histologic) has beenused as a gold standard to determinethe precise diagnostic yield of similar bronchoscopic and non-bronchoscopicprocedures.[22,28,29] However, this has a limitation that it is not useful in clinical decision making. The diagnostic utilityof this approach may be further compromised due to histologic sampling errors, the effects of previous antibiotic administration on tissuecultures, and problems related to the timing of postmortem lungexamination.[22,30] It has been suggested that the diagnostic criteria used for VAP should have high sensitivity. This approach is based on the premisethat the risk for not treating an individual patient with pneumoniaprobably outweighs the risk for unnecessary antibiotic administration.[31] For this study, we used CPIS as the standard, which has high sensitivity for the diagnosis of VAP.[5] However, there are other studies where usefulness of CPIS for the diagnosis of VAP was questioned.[32,33] Therefore, one should keep this limitation in mind during interpretation of the results of our study.

Another important limitation of our study is a relatively small cohort. Larger studies from other parts of the world have shown comparable results.[5,23,24,25,28,34,35] Indian data comparing these diagnostic modalities using independent criteria (histologic and blood orpleural fluid cultures) to establish the diagnosis of VAP are lacking; therefore, it is difficult to determine the exactoperating characteristics (sensitivity, specificity, PPV and NPV) of these non-bronchoscopic techniques. Because of this lack of established diagnostic criterion standard, we could not perform a complete economic analysis; therefore, the effect of cost associated with thetreatment of false-positive culture results cannot be commented upon.

Finally, one would expect that ETA will grow more organisms and will show false-positive results more frequently. However, our study has shown that ETA has a lower yield than NBAL, BAL and bronchoscopic brush. Although empirical broad-spectrum antibiotics might have altered the microbiologic result, however, this would have a similar effect on other microbiologic samples. This has been observed previously in antibiotic naïve as well in patients who were on empirical antibiotics.[21,34,36]

Till date, the optimal strategy for the diagnosis of VAP remains to be defined. The American Thoracic Society guidelines do provide expert opinion supporting quantitative or semi-quantitative cultures of respiratory specimens, although the panel favors invasive quantitative techniques. Our study has shown that NBAL is an acceptable alternative to bronchoscopyfor the evaluation of suspected VAP.Therefore, our observations, if verified in other ICUs, might simplify the approach for the diagnosis of VAP. This conclusion is based on the fact that NBAL is relatively inexpensive, requires lesser expertise, and may bea useful method for the serial evaluation of suspected nosocomialpneumonia in patients on mechanical ventilation.
